# Activation of α7 Nicotinic Acetylcholine Receptor Ameliorates Zymosan-Induced Acute Kidney Injury in BALB/c Mice

**DOI:** 10.1038/s41598-018-35254-1

**Published:** 2018-11-14

**Authors:** Sherehan M. Ibrahim, Muhammad Y. Al-Shorbagy, Dalaal M. Abdallah, Hanan S. El-Abhar

**Affiliations:** 1 Department of Pharmacology & Toxicology, Faculty of Pharmacy, Cairo University, Giza, Egypt; 2 School of Pharmacy, NewGiza University, Giza, Egypt; 3 FUE, Cairo, Egypt

## Abstract

Zymosan, a natural compound, provokes acute peritonitis and multiple organ dysfunction that affects the kidney, beside other organs via exaggerated inflammatory response. The aim of the present study is to test the role of cholinergic anti-inflammatory pathway (CAP) in alleviating acute kidney injury (AKI) induced by zymosan in BALB/c mice, using galantamine, a cholinesterase inhibitor, known to act via α7 nicotinic acetylcholine receptor (α7 nAChR) to stimulate CAP. Galantamine verified its anti-inflammatory effect by elevating acetylcholine (ACh) level, while abating the interleukin-6/ janus kinase 2 (Y1007/1008)/ signal transducer and activator of transcription 3 (Y705) (IL-6/ pY(1007/1008)-JAK2/ pY705-STAT3) inflammatory axis, with a consequent inhibition in suppressor of cytokine signaling 3 (SOCS3). This effect entails also the nuclear factor-kappa B (p65)/ high mobility group box protein-1/ (NF-κB (p65)/ HMGB-1) signaling pathway. Furthermore, the reno-curattive effect of galantamine was associated by a reduction in plasma creatinine (Cr), cystatin (Cys)-C, IL-18, and renal neutrophil gelatinase-associated lipocalin (NGAL), as well as an improved histopathological structure. Blocking the α7 nAChR by methyllycaconitine abolished the beneficial effect of galantamine to document the involvement of this receptor and the CAP in the amelioration of AKI induced by zymosan.

## Introduction

Galantamine (GAL), the competitive acetylcholine esterase (AChE) inhibitor, is used clinically to relief the symptoms of Alzheimer’s disease^1^. It stimulates cholinergic neurotransmission either by acetylcholine (ACh) accumulation or the allosteric binding to the α7 nicotinic acetylcholine receptor (α7 nAChR)^2^. As previously reported, GAL, the α7 nAChR agonist, enhanced the cholinergic anti-inflammatory pathway (CAP)^3^ that collaborates between the nervous and the innate immune systems^4^. Previous reports of several inflammatory disorders as diabetes^5^, rheumatoid arthritis^6^, and colitis^7^ proved the anti-inflammatory properties of GAL that are mostly mediated via the activation of the α7 nAChR^2,5,6^. Of note, one of the main mechanistic pathways by which this receptor exerts its anti-inflammatory efficacy is through the janus kinase2/ signal transducer and activator of transcription3, as well as their feedback regulator suppressor of cytokine signaling 3 (JAK2/ STAT3/ SOCS3) signaling pathway. Such pathway plays a role in regulating IL-6 and the transcriptional factor nuclear factor- kappa B (NF-κB)^7^. Moreover, NF-κB/ STAT3/ SOCS3 pathway inhibition by α7 nAChR showed a promising effect against experimental asthma^8^.

Acute kidney injury (AKI) is a serious and widely disseminated pathological condition that surprisingly has been considered as a nosocomial disease among the hospitalized patients particularly in the developed world with parallel increment in the community acquired cases^9^. Consequentially, high morbidity and mortality rates have been observed^10^. Not only the hemodynamic changes alter AKI pathophysiology but also both of inflammation and immune cells exert essential roles in kidney damage^10–12^.

Experimentally, zymosan (ZYM) induces a status of generalized inflammation^13,14^ that has deleterious effects on many organs, including the kidney^15,16^, resulting in multiple organ dysfunction syndrome (MODS)^17,18^. Such injuries are linked to the high mobility group box protein (HMGB)-1, as it exerts a key role in the inflammatory response against sterile threat induced by ZYM, as well as various infectious cases^19^ HMGB-1 is actively released in response to innate immunity activation with exogenous pathogen-derived molecules^20^. Moreover, it mediates cytokine release and tissue damage via acting as a ligand on toll like receptor (TLR)^19^. Activation of this protein is characterized by the translocation of the transcription factor NF-κB, which in turn increases its gene expression in a vicious inflammatory cycle^7^. Consequently, ZYM-induced kidney damage and HMGB-1 engenders the challenge for newly potential therapeutic targets to modulate such organs damage. Accordingly, the present work aimed to appraise the potential effectiveness of GAL against AKI evoked by sterile sepsis using ZYM in mice by targeting the CAP and to verify the involvement of the α7 nAChR in its effect.

## Material and Methods

### Animals

Adult 8 weeks old male BALB/c mice weighing 22–25 g were purchased from El Nile Pharmaceutical Company (Cairo, Egypt). Animals were kept to accommodate in the facility of the Faculty of Pharmacy, Cairo University (Cairo, Egypt) a week before carrying the experiment. Mice were maintained under controlled temperature (24 ± 2), a 12 h day/night cycle, and were fed standard chow diet and tap water ad libitum.

### Compliance with Ethical standards

This study was carried out in accordance with the recommendations of the ethical standards of Guide for the Care and Use of Laboratory Animals (No. 86-23; NIH Publications, 1996) and the protocol was approved by the Research Ethics Committee of Faculty of Pharmacy, Cairo University (Cairo, Egypt) with the permit number PT 1450.

### Experimental design

Animals were divided into four groups (n = 10, each); mice in the 1^st^ group received saline to serve as the control group, whereas those in the other three groups (groups 2–4) were injected with ZYM (750 mg/kg; i.p)^21^. This dose of ZYM was selected according to a preliminary pilot experiment that showed a reasonable renal damage with 13% mortality rate (2/15 mice) after 24 h of ZYM challenge in ZYM group. Group 2 presented the untreated control model, where mice received only saline, while in group 3 animals were injected with GAL (4 mg/kg; i.p)^22^ at 1 and 6 h after single ZYM injection. Mice in the last group received the blocker methyllycaconitine (MLA; 5.6 mg/kg; i.p)^23^ 15 min before each dose of GAL to serve as the ZYM + MLA + GAL group.

### Blood and tissue sampling

After 24 h of ZYM injection, animals were anaesthetized and blood was collected in heparinized tubes from inferior vena cava for plasma separation. Mice were then euthanized and the kidneys were harvested and kept in the corresponding media. For ELISA, the right kidneys of 6 mice were homogenized in phosphate buffer saline (PBS) and 3 left kidneys were kept in RIPA buffer (Bio BASIC, Ontario, Canada) for parameters assessed by Western blot. In the remaining 4 animals, the right kidneys were kept in 10% PBS-buffered formalin for the histopathological examination, whereas the left kidneys were kept in RNA later solution (Thermo Fisher Scientific, MA, USA) for RT-PCR evaluation.

### Assessed parameters

For detection of ZYM-induced AKI and the effect of GAL, a histopathological examination was carried out in addition to the measurement of renal biomarkers; viz., plasma creatinine (Cr), interleukin-18, cystatin (Cys)-C, as well as renal neutrophil gelatinase-associated lipocalin (NGAL). Furthermore, to study the possible and involved mechanism(s) of how GAL may act against AKI, acetylcholine (ACh) was assessed besides the inflammatory trajectory IL-6/ pY1007/1008-JAK2/ pY705-STAT3/ SOSC3. Besides, the renal transcriptional factor, NF-κB (p65), and the expression of one of its downstream molecules, HMGB-1 that acts in a vicious cycle were measured. These parameters were further evaluated to highlight their role in kidney damage induced by ZYM.

### Techniques

#### Colorimetric method (End point)

Plasma Cr (Biodiagnostic colorimetric kit, Cairo, Egypt) was measured according to the kit manufacturers’ instructions. The principle depends on the formation of a colored complex between Cr and picrate in alkaline medium.

#### ELISA technique

Plasma ACh (EIAab, Wuhan, PRC, Cat. # E0912m), IL-6, IL-18, and Cys-C (Cusabio, Wuhan, PRC, Cat. # E04639m, E04609m, and E08386m, respectively) were assessed. In kidney homogenate, NGAL as well as NF-κB (p65) (Cusabio, Wuhan, PRC, Cat. # E09410m and E08789m, respectively) were measured. All ELISA experiments procedures comply with the manufacturers’ instructions.

#### Western blot analysis

The kidney lysate aliquots were brought to complete protein extraction, after several processing steps and the protein concentration was determined using Bradford Protein Assay Kit (Bio Basic, Ontario, Canada). The protein concentration of each sample was loaded with an equal volume of 2x Laemmli buffer (125 mM Tris, pH 6.8; 10% glycerol, 10% SDS; 0.006% bromophenol blue; 130 mM DTT), then the mixture was boiled for 90 sec at 100 °C. Protein samples were loaded and separated by 10% SDS-PAGE (Bio-Rad, CA, USA) using mini protein electrophoresis separation unit (Bio-Rad, CA, USA). This was followed by gel electrophoresis transfer onto polyvinylidenedifluoride (PVDF) membranes using Trans-Blot Turbo instrument (Bio-Rad, CA, USA).The membranes were blocked at room temperature for 1 h using blocking buffer (20 mM Tris, pH7.5; 150 mM NaCl; 0.1% Tween 20; 3% bovine serum albumin), then the blocked blots were incubated overnight at 4 °C with the primary antibodies; anti-pY705-STAT3 (1:1000), anti- pY1007/1008-JAK2 (1:1000) (Invitrogen, CA, USA), and anti-suppressor of cytokine signaling (SOSC)3 (1:200) (Thermo Fisher Scientific, MA, USA) as well as the antibody of the loading control anti- β-actin antibody (1:1000) (Santa Cruz Biotechnology, CA, USA). The latter was probed to assure equivalent sample loading, then the blot was washed five times in a mixture of Tris-buffered saline with Tween 20. This was followed by incubating the blot membranes to HRP conjugated to anti-rabbit antibody (Dianova, Hamburg, Germany). The charge coupled device (CCD) camera-based imager was used to capture the chemiluminescent signals and image analysis software was used to read the band intensity of the studied proteins on the Chemi Doc MP imager (Bio-Rad, CA, USA). Results were expressed as arbitrary units (AU) after normalization for β-actin protein expression.

#### Quantitative RT-PCR analysis

Total RNA was extracted from the kidney using RNeasy mini kit (Qiagen, MD, USA). All procedures were done according to the manufacturer protocol. Any residual DNA was removed by the kit provided DNase. The concentration of the isolated RNA in each sample was measured at 260 nm using spectrophotometer and the purity of isolated RNA was assessed by absorption ratio at 260/280 nm. For cDNA synthesis, a reverse transcription system was used (Fermentas, MA, USA), where the RNA was incubated with 5X first strand reverse transcription buffer, 10 mM dNTP mixture, oligo d (t) primers, 40U/µl RNase inhibitor, and 50 U/µl MMLV-RT enzyme at 42 °C for 60 min. The PCR reactions include 10 min at 95 °C for activation of AmpliTaq DNA Polymerase, followed by 40 cycles at 94 °C for 15 sec (denaturing), 60 °C for 1 min (annealing), and 72 °C for 30 sec (extension) using Applied Biosystem with software version 3.1 (Thermo Fisher Scientific StepOne™, MA, USA). The quantitative PCR reaction mixture consisted of SYBR green, cDNA template, RNase free water, in addition to the sequences of forward and reverse primers for high mobility group box protein (HMGB)-1 and β-actin (Table 1). The relative quantification was calculated from the 2^−ΔΔCT^ formula^24^ using *β*-actin as the internal standard genes.

**Table 1:** The primer sequence of the HMGB-1 and β-actin genes.

	Primer sequence	Gene bank accession number
HMGB-1	Forward primer: 5′-TCAATTCTGTCACACCATGGGA-3′ Reverse primer: 5′-AAGCTCACGCTTTTGGGGAT-3′	NM_012963.2
β-actin	Forward primer :5′-GGTCGGTGTGAACGGATTTGG-3′ Reverse primer:5′-ATGTAGGCCATGAGGTCCACC-3′	XM_017593963.1

#### Histopathological examination

The samples were fixed with 10% PBS buffered formalin for 8 h at room temperature, embedded in paraffin, and sectioned to 4 *μ*m thickness. After deparaffinization and rehydration, the sections were stained with hematoxylin and eosin (H&E). The severity of renal damage was semiquantitatively assessed^15,25^ with few modifications. The degree of renal injury was scored based on a subjective scale ranging from 0 to 3, where 0 = absence, 1 = mild, 2 = moderate, and 3 = severe. The ranging scale was used for vacuolation of renal tubules, epithelium congestion, glomerular tuft congestion of renal blood vessels, atrophy of glomerular tuft, necrosis of epithelial lining, and renal tubules perivascular inflammatory cells infiltration. The histological evaluations were done by a single investigator in a blinded manner and the renal damage grading was measured as an average score of the score mean of each criteria.

### Statistical Analysis

Results are expressed as mean ± SD. The GraphPad Prism v5.0 (GraphPad Prism, CA, USA) was used to analyze and express all the available data. For multiple comparisons, one-way analysis of variance (ANOVA), followed by Tukey post-hoc test was used. For histopathological scoring, significance was maintained only while using non-parametric Mann–Whitney U test. P < 0.05 was considered as the significance limit for all comparisons, unless otherwise stated.

## Results

### GAL via α7 nAChR ameliorates kidney dysfunction in ZYM-induced AKI

**Figure 1:**
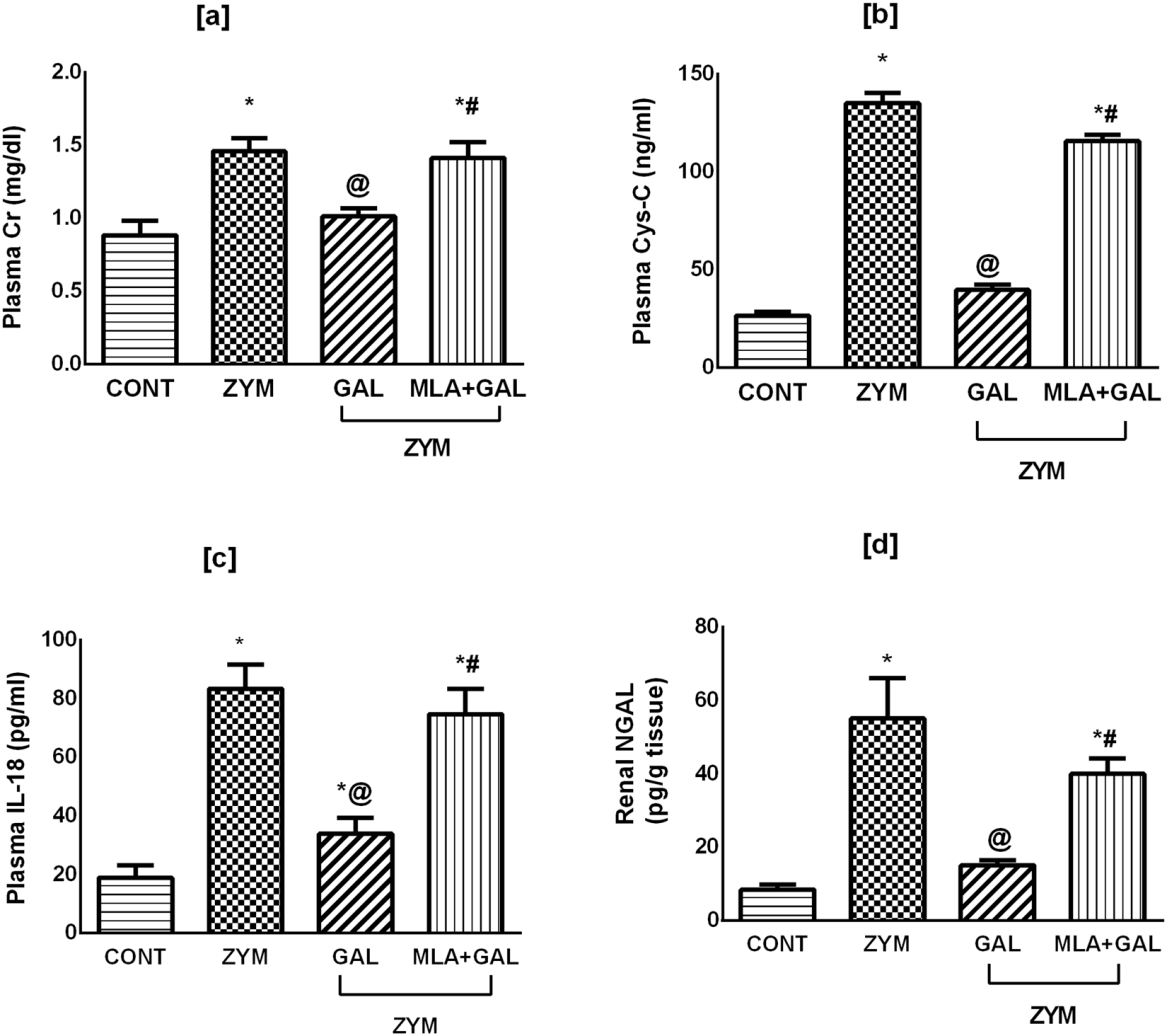
Effect of GAL (4 mg/kg; i.p) and/or MLA (5.6 mg/kg; i.p) on plasma levels of [a] Cr, [b] Cys-C and [c] IL-18, as well as renal [d] NGAL content in ZYM-induced AKI. Data are expressed as mean of 6 mice ± SD. Statistical analysis was performed using one way analysis of variance (ANOVA) followed by Tukey’s Multiple Comparison test; as compared to CONT(*), ZYM (^@^), and ZYM + GAL (^#^) groups, P < 0.05. GAL was injected 1 and 6 h post ZYM challenge, while MLA was administered 15 min before GAL. AKI: acute kidney injury; CONT: control; Cr: creatinine; Cys-C: cystatin C; GAL: galantamine; MLA: methyllycaconitine, IL-18: interleukin-18; NGAL: neutrophil gelatinase-associated lipocalin; ZYM: zymosan.

As depicted in Fig. 1, the current model significantly elevated the plasma levels of [a] Cr (*p* < 0.05), and boosted that of [b] Cys-C, [c] IL-18, and renal [d] NGAL (*p* < 0.001), compared to the normal control group. The post-administration of GAL moderately reduced plasma [a] Cr (*p* < 0.05) and sharply abated other biomarkers (p < 0.001) increments to provide its effectiveness in ameliorating AKI in ZYM challenging model. On the other hand, the use of α7 nAChR antagonist MLA abolished the GAL favorable effects on the kidney.

### GAL via α7 nAChR modulates plasma ACh and the proinflammatory mediators in ZYM-induced AKI in mice

**Figure 2:**
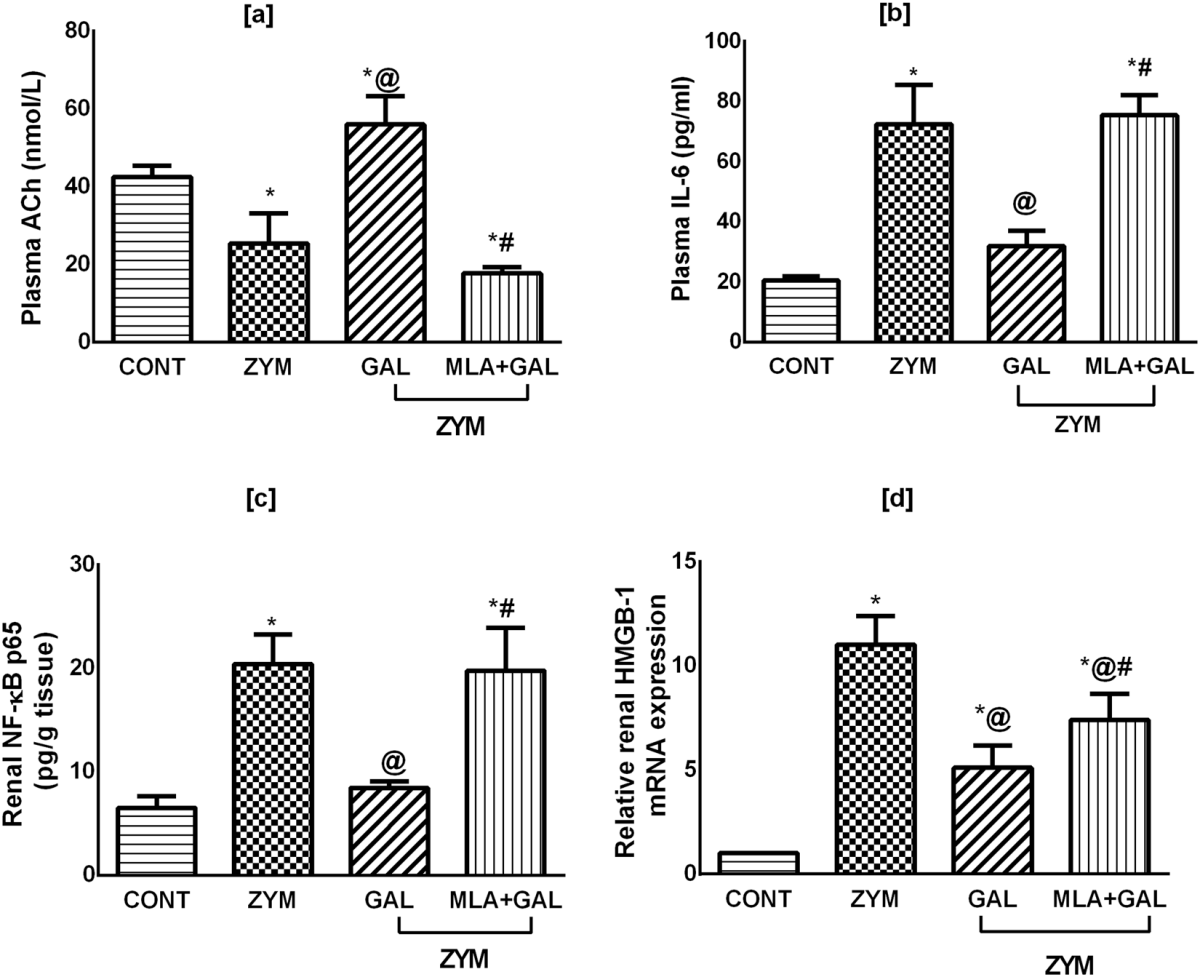
Effect of GAL (4 mg/kg; i.p) and/or MLA (5.6 mg/kg; i.p) on plasma levels of [a] ACh, [b] IL-6, as well as renal contents of [c] NF-κB (p65) and [d] HMGB-1 in ZYM-induced AKI. Data are expressed as mean of 6/4 mice ± SD. Statistical analysis was performed using one way analysis of variance (ANOVA) followed by Tukey’s Multiple Comparison test; as compared to CONT(*), ZYM (^@^), and ZYM + GAL (^#^) groups, P < 0.05. GAL was injected 1 and 6 h post ZYM challenge, while MLA was administered 15 min before GAL. ACh: acetylcholine; AKI: acute kidney injury; CONT: control; GAL: galantamine; HMGB: high mobility group box protein; MLA: methyllycaconitine; IL-6: interleukin-6; NF-κB: nuclear factor-kappa B; ZYM: zymosan.

Figure 2 shows that ZYM model markedly depleted the plasma level of [a] ACh, but boosted that of [b] IL-6. In the kidney homogenate; the same pattern was observed, where ZYM highly increased [c] NF-κB content and upregulated the [d] HMGB1 gene expression (*p* < 0.001). These perturbations were markedly corrected in the GAL post-treated group (p < 0.001), while the administration of MLA before GAL reverted plasma ACh, IL-6, and NF-κB (*p* < 0.001) to be comparable to that in the ZYM untreated group associated with a significant reduction in HMGB1 (*p* = 0.0455).

### GAL via α7 nAChR alters kidney pY1007/1008-JAK2, pY705-STAT3, as well as SOCS3 in ZYM-induced AKI

**Figure 3:**
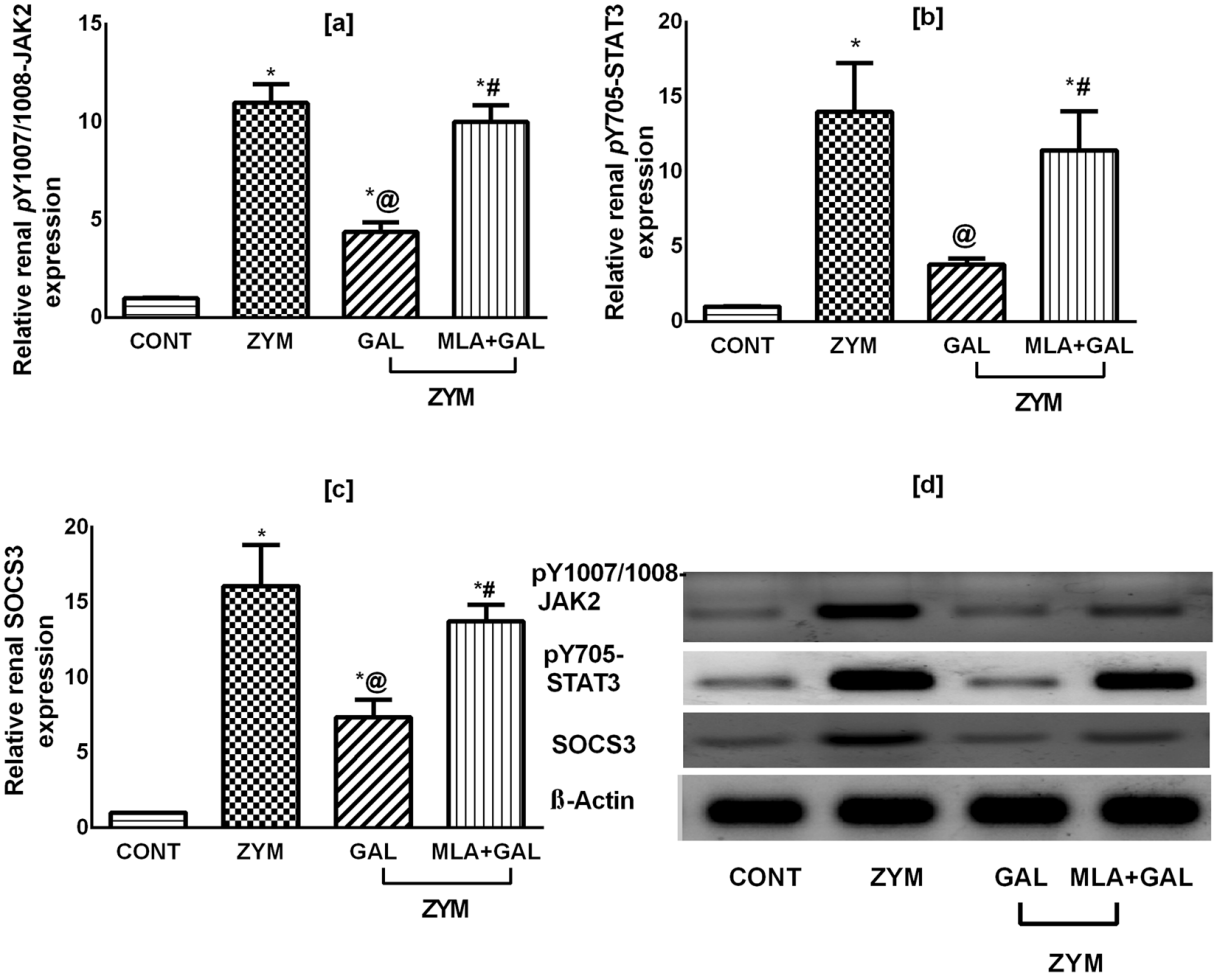
Effect of GAL (4 mg/kg; i.p) and/or MLA (5.6 mg/kg; i.p) on protein expression of [a] *p*-JAK-2, [b] *p-*STAT-3, and [c] SOCS3, as well as their corresponding [d] cropped blots in ZYM-induced AKI. Data are expressed as mean of 3 mice ± SD. Statistical analysis was performed using one way analysis of variance (ANOVA) followed by Tukey Multiple Comparison test; as compared to CONT(*), ZYM (^@^), and ZYM + GAL (^#^) groups, P < 0.05. GAL was injected 1 and 6 h post ZYM challenge, while MLA was administered 15 min before GAL. AKI: acute kidney injury; CONT: control; GAL: galantamine; MLA: methyllycaconitine, *p*-JAK: phosphorylated janus kinase (Y1007/1008); *p*-STAT: phosphorylated signal transducer and activator of transcription (Y705); SOCS: suppressor of cytokine signaling; ZYM: zymosan. β-Actin was used as the housekeeping reference protein.

Relative protein expression of the pY1007/1008-JAK2, pY705-STAT3, and SOCS3, in reference to the housekeeping protein β-actin, were markedly enhanced in the ZYM group by 11, 14, and 16 folds, respectively, compared to the control group (Fig. 3a–c, respectively; *p* < 0.001); GAL, on the contrary, reverted these effects. Noteworthy, ZYM + MLA + GAL group eradicated the effects mediated by the cholinergic agonist. The administered drugs showed extreme significant alterations in these parameters (*p* < 0.001). Supplementary data for western blotting analysis are available.

### GAL via α7 nAChR improves renal morphological alterations in ZYM-induced AKI

**Figure 4:**
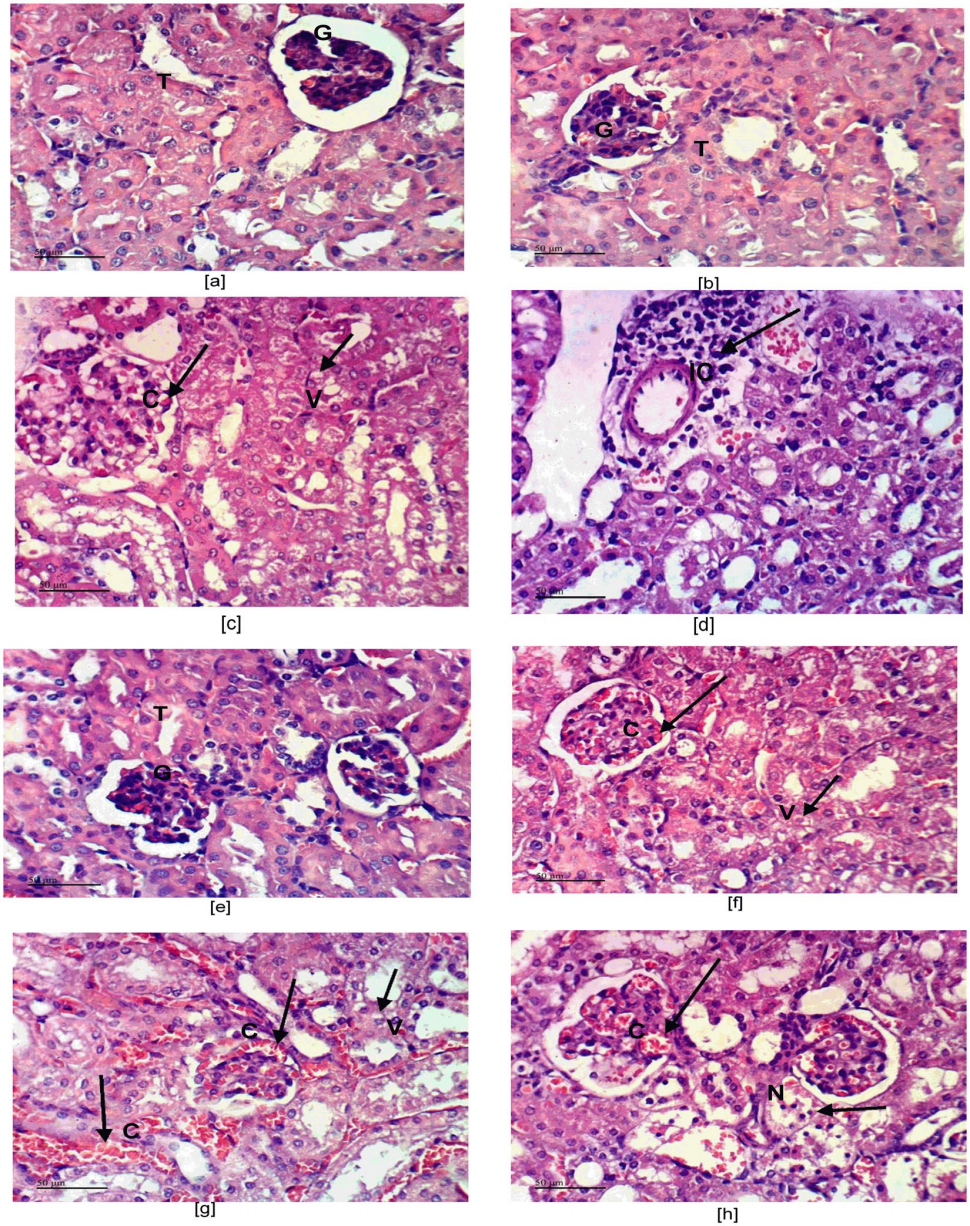
Effect of GAL (4 mg/kg; i.p) and/or MLA (5.6 mg/kg; i.p) on renal histopathological changes in ZYM-induced AKI. Sections of ZYM treated animals show [c] cytoplasmic vacuolization (V) of epithelial lining renal tubules, congestion (C), and hypercellularity of glomerular tuft (large arrow), besides [**d**] perivascular inflammatory cells (IC) infiltration, compared to [a and b] CONT mice showing the normal histological structure of renal tubules (T) and glomeruli (G). Sections of GAL show [e] no histopathological changes in both renal tubules (T) and glomeruli (G) or [f] slight cytoplasmic vacuolization (V) and slight congestion (C) of glomerular tuft. Sections of MLA + GAL show [g] cytoplasmic vacuolization (V) of epithelial lining of some renal tubules, with congestion (C) of glomerular tuft and renal blood vessels, in addition to [h] necrosis (N) of epithelial lining renal tubules (H&E X 400).

**Figure 5:**
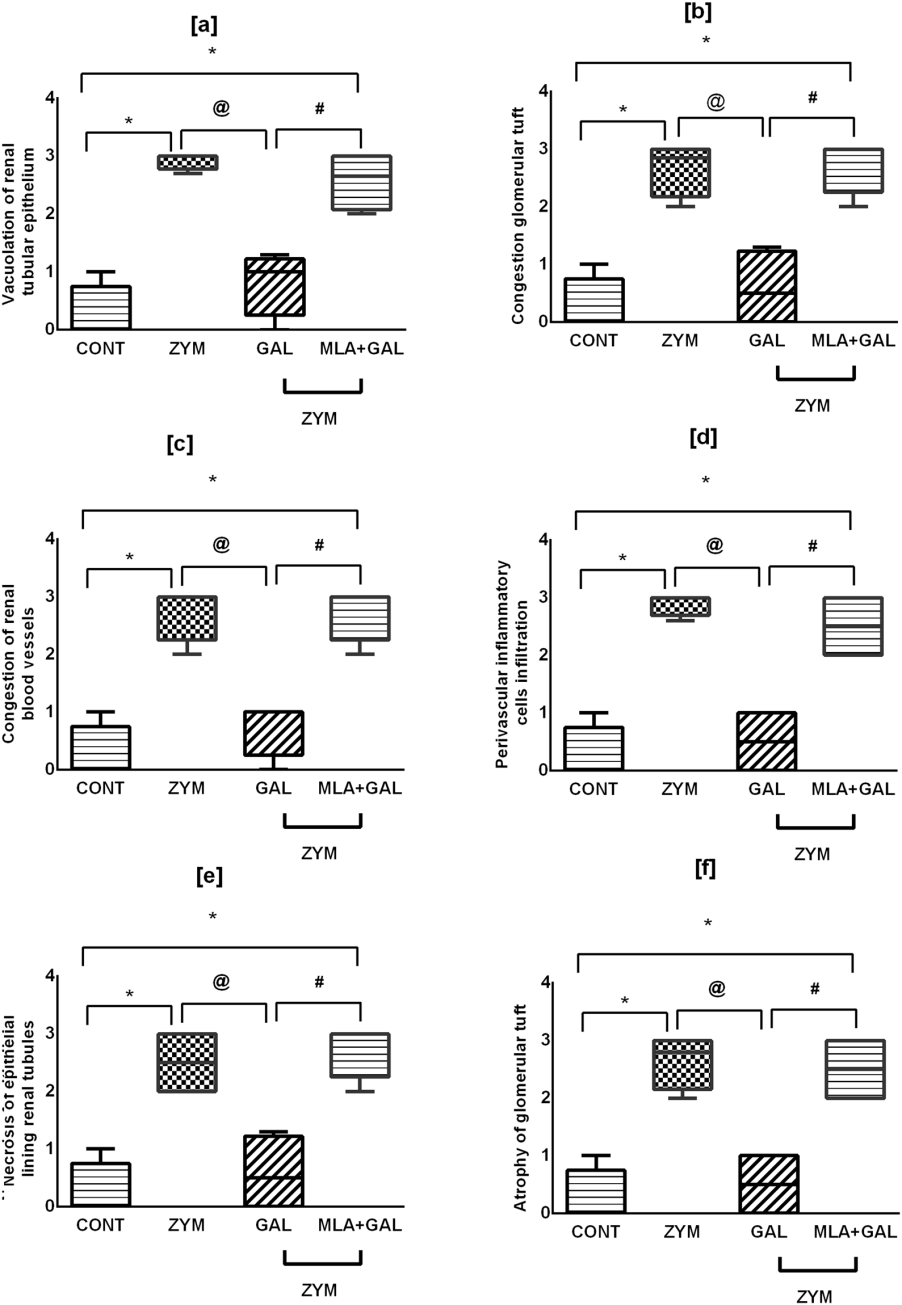
Histopathological renal score [a–f]. For histopathological scoring, results (n = 4 mice per group) are expressed as boxplots with median (minimum to maximum). Comparison was held using non-parametric Mann–Whitney U test; as compared to CONT (*), ZYM (^@^), and ZYM + GAL (^#^) groups, P < 0.05. GAL was injected 1 and 6 h post ZYM challenge, while MLA was administered 15 min before GAL. AKI: acute kidney injury; CONT: control; GAL: galantamine; MLA: methyllycaconitine; ZYM: zymosan.

Photomicrograph sections (Fig. 4) show [a and b] normal renal parenchyma architecture in control untreated mice, whereas [c and d] ZYM sections displayed cytoplasmic vacuolization of epithelial lining renal tubules, glomerular tuft congestion and hypercellularity with perivascular inflammatory cells infiltration. Moreover, sections [e and f] of GAL treated group show slight cytoplasmic vacuolization of some epithelial lining renal tubules with minor congestion of glomerular tuft. Sections of (g and h) MLA + GAL treated animals display epithelial lining cytoplasmic vacuolization with glomerular tuft dilatation and congestion, as well as necrosis of renal blood vessels of epithelial lining renal tubules. Fig. 5 [a–f] summarizes the aforementioned data represented by histopathological renal score.

## Discussion

**Figure 6:**
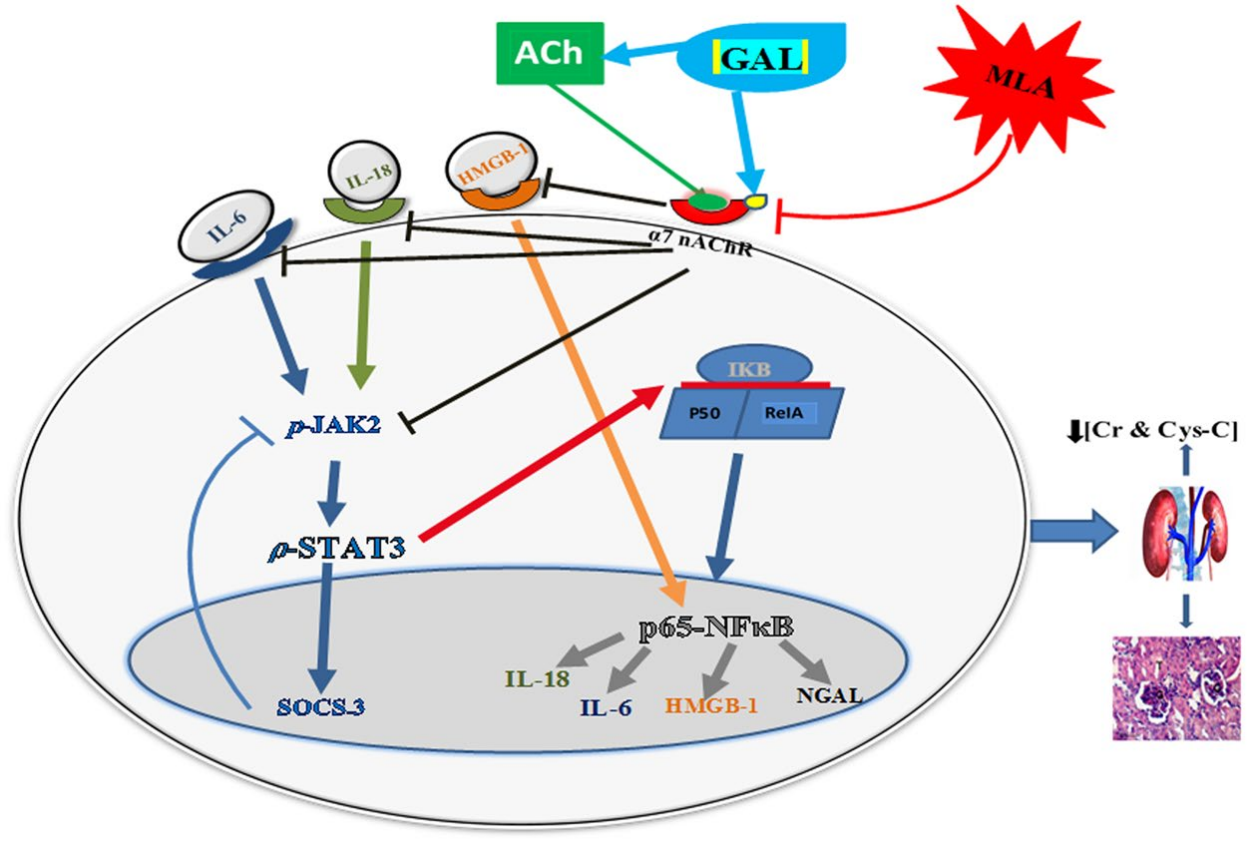
Schematic pathway summarizing the manipulation of GAL *via* the activation of α7 nAChR to mediate its anti-inflammatory effect. GAL mediated its anti-inflammatory effect by inhibiting the IL-18-IL-6/ p-JAK2/ p-STAT3/ SOCS3 and NF-κB/ HMGB-1/ IL-18 trajectories. This was associated with improvement in kidney function (Cr, Cys-C, IL-18, NGAL) and histological structure. The beneficial renocurative effect of GAL was abolished by the use of MLA in ZYM-induced AKI in BALB/c mice. SOCS3 works as negative feedback pathway to inhibit IL-6/ STAT3, while here GAL inhibited SOCS3 possibly due to the ability of GAL to directly inhibit IL-6, which does not necessitate the activation of SOCS3. ACh: acetylcholine; AKI: acute kidney injury; CAP: cholinergic anti-inflammatory pathway; Cr: creatinine; Cys-C: cystatin-C; GAL: galantamine; HMGB-1: high motility group box-1; IκB: inhibitor of κB; IL: interleukin; JAK2: janus kinase 2; MLA: methyllycaconitine; α7 nAChR: α7 nicotinic acetylcholine receptor; NGAL: neutrophil gelatinase-associated lipocalin; p65 NFκB: p65 nuclear factor kappa B; STAT3: signal transducers and activators of transcription; SOCS: suppressor of cytokine signaling; Zym: zymosan.

The present study highlighted the involvement of CAP, via activation of the α7 nAChR, in the reno-protective effect of GAL against a ZYM-induced kidney injury model. Post administration of GAL ratified its anti-inflammatory potential by elevating ACh level and inhibiting the IL-6/ p-JAK2/ p-STAT3/ SOCS3 pathway, besides abating the HMGB-1/ NF-κB (p65) inflammatory vicious cycle. The favorable modulatory effects of these pathways were further confirmed by the marked decrease in the kidney function tests, *viz*., Cr, Cys-C, IL-18, and NGAL, in addition to the improvement of histopathological alterations. Since the selective blocker, MLA, abolished GAL effects at all levels, hence it could be concluded that GAL anti-inflammatory mechanisms are mediated through α7 nAChR. These effects were summarized and displayed in Fig. 6 as a schematic pathway.

Currently, vagal stimulation has been documented to have a beneficial effect in modulating several inflammatory conditions^26^. Based on a series of studies^27,28^, GAL anti-inflammatory action was reported to override that of other anti-cholinesterases, by virtue of its allosteric modulation of α7 nAChR to synergistically activate the CAP. The findings of the present study concur with this fact, where GAL elevated ACh level, besides activating the α7 nAChR. The latter effect was confirmed by using the selective α7 nAChR blocker, which abolished its favorable effects.

GAL mediated its anti-inflammatory effect via an interplay between constellations of cues. In the present work, GAL attenuated ZYM-induced IL-6, which can be owed to the elevated level of ACh; previously, ACh was reported to reduce the release, expression, and/or mRNA half-life of IL-6^29,30^. This inhibition entailed the downstream JAK2/ STAT3 cascade, for IL-6 being the initiator of this pathway^31^. In accordance to our results, activated/phosphorylated JAK2/ STAT3 axis was responsible for the injurious effect of different AKI insults^32,33^. Hence, the ability of GAL to suppress this signal was a culprit for the improved kidney function assessed here, besides its ability to stimulate the α7 nAChR. Ample of evidence have highlighted the importance of this signal in alleviating several inflammatory conditions via stimulating the α7 nAChR. Suppression of p-JAK2/ p-STAT3 by their inhibitors^34^ or the use of cholinergic agonists^34–36^ in LPS models was the corner stone in alleviating inflammation. These authors ascribed the blunted p-JAK2/ p-STAT3 axis to the activation of α7nAChR. In a ZYM-induced AKI model, Dimitrova *et al*.^15^ emphasized the role of this pathway in improving kidney function upon using a JAK2 inhibitor^15^, to support further the current results. ZYM-induced AKI also elevated SOCS3, which is the downstream feedback molecule that is released in attempt to brake the inflammatory pathway IL-6/ p-JAK2/ p-STAT3. Nevertheless, in the GAL treated group, SOCS3 was also decreased possibly because of the attenuated pathway and/or the anti-inflammatory potential of GAL that does not demand the production of the feedback molecule as reported herein. A similar result was documented before in an *in-vitro* model using nicotine^37^. The present study proved that activation of α7 nAChR was the one responsible for the inhibition of the IL-6/ JAK2/ STAT3/ SOCS3, where the GAL effect was completely blocked upon using the selective antagonist MLA.

IL-6 is known to be one of the pro-inflammatory mediators transcribed by the transcription factor NF-κB (p65). This factor was revoked by the post-administration of GAL again by virtue of curtailing the p-JAK2/ p-STAT3 axis. In harmony with our findings, previous studies^34,36^ have conveyed that upon curbing the p-JAK2/ p-STAT3 signal, the unphosphorylated STAT3 (U-STAT3) is enhanced to compete with IκBα, hence, sequestering NF-κB (p65) and limiting its activation, translocation into nucleus, and its inflammatory response. HMGB-1 is another NF-κB (p65)-related downstream pro-inflammatory mediator^38^ that increases during sepsis and generalized inflammatory conditions^20^, as was shown in the present work. Moreover, in a positive feedback loop, HMGB-1 acts as a ligand for either TLR or RAGE to enhance the NF-κB signaling pathway^39–41^. Therefore, GAL-induced inactivation of NF-κB is responsible, in part, for the inhibition of HMGB-1, besides IL-6 to signify its anti-inflammatory potential. This effect relies on the increased release of ACh and the activation of α7 nAChR, where MLA obliterated the GAL curative effect.

Additionally, another inflammatory cytokine that has been inhibited by GAL is IL-18; the cytokine is an important mediator of renal tubular epithelial cell injury that was boosted in the ZYM model. In traversing routes, IL-18 upon binding to its receptor deepened the ZYM-induced inflammatory/injurious effect *via* activating the JAK2/STAT3/SOCS3 pathway^42^, stimulating the transcription factor NF-κB^43^, and increasing the production of IL-6 among other cytokines^44,45^, to trigger several inflammatory cascades. GAL effect can be linked to the elevated level of ACh, as proven herein and previously^29^, as well as to the activation of the cholinergic receptor confirmed by the MLA-induced limitation of GAL effect. The ability of GAL to lower SOCS3 can be linked to the suppression of IL-18 as suggested by Matsui *et al*.^42^; these authors revealed that SOCS3 may be increased as a result of the increased IL-18 content and/or to regulate the activated STAT-3 pathway^44^. Additionally, upon using a p-STAT3 inhibitor (S3I-201), the authors recorded decreases in IL-18 and SOCS3, as well.

Apart from producing inflammatory cytokines, NF-κB also modulates NGAL, which is a protein that is up-regulated in several injury settings^46–48^ and is linked to numerous cellular responses^49^. NF-κB was reported to be essential for NGAL expression^50^, an effect that can explain its inhibition in the GAL treated group as a consequence to the inactivation of NF-κB.

## Conclusion

Depending on the results of the current study, GAL post-treatment improved kidney function through acting on the α7 nAChR that modulates the inflammatory pathways; *viz*., IL-6/ JAK2/ STAT3/ SOCS3 and NF-κB (p65)/ HMGB-1/ IL-18 that collaborate to induce AKI. This conclusion was supported by the administration of the α7 nAChR blocker MLA, which abolished the reno-curative effect of GAL. The study paves the way for upcoming experimental studies and nominates GAL as a useful therapy for future studies in AKI clinically.

## Electronic supplementary material

Supplementary data for western blotting

## Data Availability

All data generated or analysed during this study are included in this published article and its supplementary information files.
