# Supplementary material for: Activation of α7 Nicotinic Acetylcholine Receptor Ameliorates Zymosan-Induced Acute Kidney Injury in BALB/c Mice
Source: Sci Rep. 2018 Nov 14;8:16814. doi: 10.1038/s41598-018-35254-1 (PMC6235888; doi:10.1038/s41598-018-35254-1)
Supplement: Supplementary file 1 — Supplementary data for western blotting [file 41598_2018_35254_MOESM1_ESM.pdf]

Activation of α7 Nicotinic Acetylcholine Receptor Ameliorates Zymosan-Induced Acute Kidney Injury in BALB/c mice

Sherehan M. Ibrahim [a]*, Muhammad Y. Al-Shorbagy [a,b], Dalaal M. Abdallah [a], Hanan S. El-Abhar [a]

[a] Department of Pharmacology & Toxicology, Faculty of Pharmacy, Cairo University, Egypt

[b] School of Pharmacy, NewGiza University, Egypt

* Correspondence:
Sherehan M. Ibrahim, Sherehan.mohamed@pharma.cu.edu.eg, Tel: +201003827007

*p*Y1007/1008 - JAK2 | *p*Y705-STAT3 | *SOCS3*

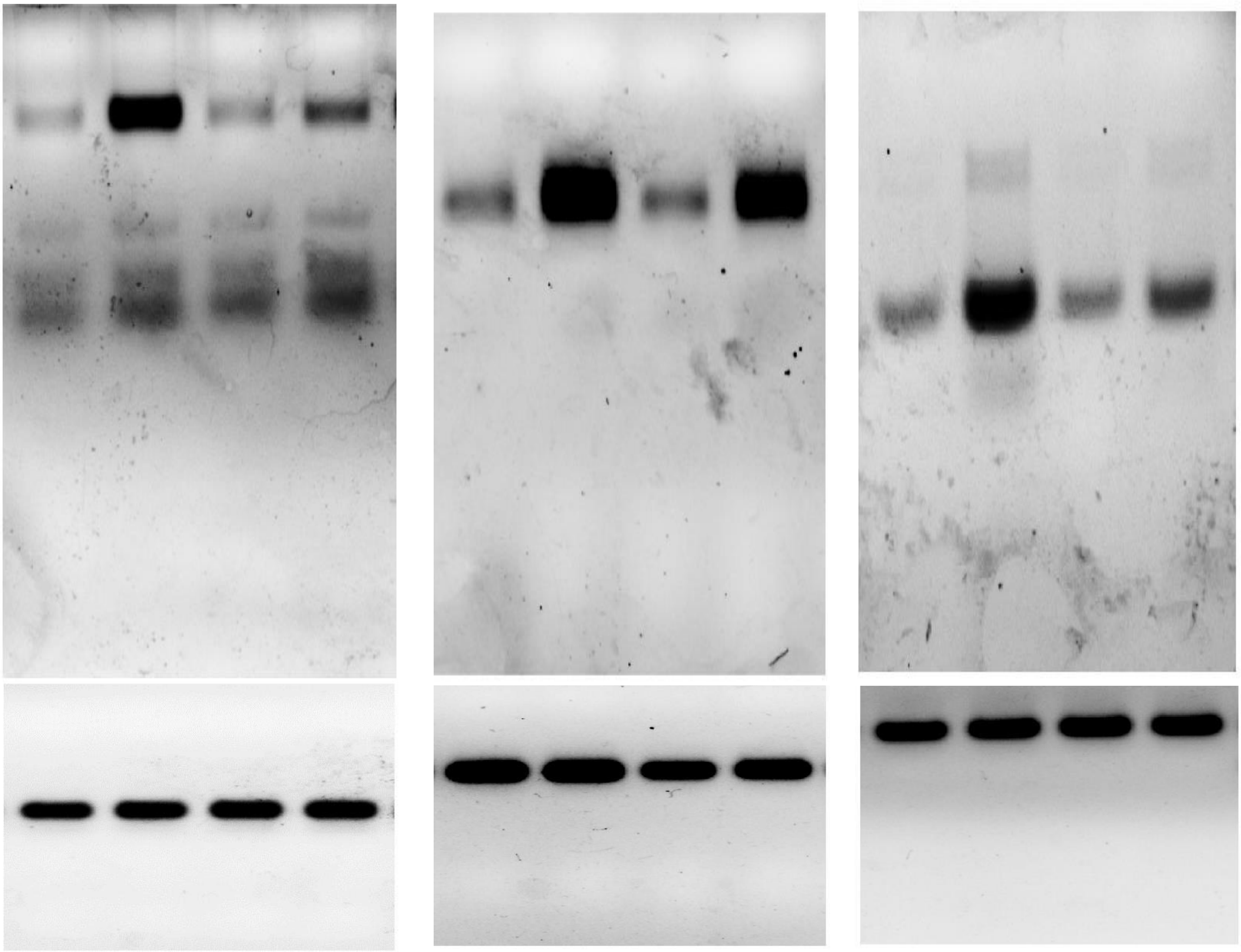

β-Actin | *β-Actin* | *β-Actin*
